# A Study on Online Health Community Users’ Information Demands Based on the BERT-LDA Model

**DOI:** 10.3390/healthcare11152142

**Published:** 2023-07-27

**Authors:** Minhao Xiang, Dongdong Zhong, Minghua Han, Kun Lv

**Affiliations:** Business School, Ningbo University, Ningbo 315211, Chinazhongdongdong@nbu.edu.cn (D.Z.);

**Keywords:** information demands, sentiment analysis, health information, text mining, online health communities

## Abstract

As the economy and society develop and the standard of living improves, people’s health awareness increases and the demand for health information grows. This study introduces an advanced BERT-LDA model to conduct topic-sentiment analysis within online health communities. It examines nine primary categories of user information requirements: causes, symptoms and manifestations, examination and diagnosis, treatment, self-management and regulation, impact, prevention, social life, and knowledge acquisition. By analyzing the distribution of positive and negative sentiments across each topic, the correlation between various health information demands and emotional expressions is investigated. The model established in this paper integrates BERT’s semantic comprehension with LDA’s topic modeling capabilities, enhancing the accuracy of topic identification and sentiment analysis while providing a more comprehensive evaluation of user information demands. This research furthers our understanding of users’ emotional reactions and presents valuable insights for delivering personalized health information in online communities.

## 1. Introduction

As the living standard improves and the internet develops rapidly, people’s health management awareness increases, resulting in a growing demand for online health information access. Statistics indicate that by December 2021, China had 298 million internet medical users, with a substantial proportion utilizing online platforms for medical and health services [1]. Approximately 80% of internet users search for health-related information online, 34% browse evaluations and experiences concerning health and medical issues in online communities, and 24% seek information about drug efficacy and medical diagnosis online. Considering the significant challenges facing China’s healthcare system, such as medical resources shortage, an aging population, and a high disease burden [2,3], online health communities have emerged as vital health information and support sources for patients and caregivers [4]. Online health community users have diverse health information demands, encompassing disease prevention, treatment options, and self-management [5]. However, obtaining accurate and reliable health information can be challenging, especially in China’s rapidly evolving healthcare landscape [6]. As the volume of online health information grows, individuals increasingly struggle to access specific health knowledge from such a vast and complex resource pool [7,8]. This creates a conflict between the abundance of available health information and the demand for accurate, reliable knowledge. Consequently, developing a more precise method for identifying and analyzing online community users’ information demands is crucial to accurately match users’ health information demands and available health information supply.

Online health communities, as platforms for users to search for health information, seek health assistance, and share experiences, have accumulated vast amounts of communication content and user behavior data in textual form. This provides a foundation for investigating and developing online health information demands. Presently, both domestic and international scholars are focusing on studying the online health community information demands field, primarily centered on identifying user information demand [9], extracting user information demand characteristics [10], constructing a health information Question and Answer (Q&A) quality evaluation system [11], and exploring the triggering mechanisms and influence factors of user question behavior [12]. Based on the developmental history of research methods and technical approaches, the field of online health information demands has predominantly experienced two crucial stages: intervention-based and non-intervention-based. Initially, due to the limited availability of internet medical health information resources, we primarily employed intervention-based research approaches to understand user experiences and perspectives, both domestically and internationally. Through visits, online health community users, and surveys, direct user demands and experiences were obtained [13]. However, this research method is susceptible to influences such as the sample data and interviewees, making it difficult to reflect the entire user group’s information demands. With the emergence of large online health platforms, research methods have shifted towards non-interventional approaches. This type of research relies on data provided by large online health platforms [14] and utilizes methods such as grounded theory [15], content analysis [16], structural equation modeling [17], and multiple regression analysis [18] to conduct research. This offers a more objective reflection of user information demands and a more scientific basis for improving health information services quality. Additionally, empirical research on online health information demands and other related information behaviors has become increasingly diverse across various theoretical perspectives and application contexts. Scholars primarily focus on online health communities and social media, exploring the influence of individual factors, such as motivation, on willingness to acquire and share health information. They employ theories such as social capital theory, social cognitive theory, planned behavior theory, and technology acceptance models [19,20,21,22]. Regarding the investigation of user emotional expression demands and characteristics, some scholars employed correlation analysis to examine the association between the size of online health communities and the emotional expression of HIV/AIDS patients [23]. They discovered a weak positive correlation between the proportion of users exhibiting negative emotions in each community and community size. On the other hand, some researchers have utilized Facebook data to analyze user emotional expression characteristics, identifying types of users who require emotional support and discussing the positive impact of such support [24,25]. Sentiment tendencies within the text are analyzed to study the relationship between users’ health demands and emotional demands, which is crucial for enhancing information retrieval efficiency and explaining information behavior [26].

With the widespread adoption and development of the internet, the availability and quantity of medical health information have increased exponentially. This surge has led to information redundancy. The same content and advice are repeatedly mentioned across different websites and platforms, sometimes even presenting contradictory viewpoints, causing difficulties for users seeking effective information. To address this issue, researchers have leveraged the large volume of posts and user behavior data within online communities to extract user characteristics and investigate information demands. Quantitative methodologies, including manual coding and statistical analysis, are employed to uncover user features and information requirements. Techniques such as K-means clustering [27], EM clustering [28], and natural language processing-based text mining [29] enable more comprehensive and precise topic discovery and recognition. Furthermore, some research focuses on implementing automatic diagnosis and intelligent medical services in online health communities. By using text classification techniques like support vector machines and conditional random fields, researchers aim to match user information demands accurately through topic recognition and domain entity identification within text data [30,31]. Among these, topic modeling, a topic recognition method, employs the Latent Dirichlet Allocation (LDA) model to transform text data into topic probability distributions, revealing users’ attention levels and emotional tendencies towards various topics [32]. However, as research advances and new methodologies emerge, limitations of traditional topic modeling methods, such as the LDA model, have become evident. For instance, LDA models may produce topics with overlapping words, resulting in ambiguous interpretations [33], and may not accurately capture sentiment or emotions expressed in the text [34]. These limitations offer opportunities for improvement in capturing and analyzing the information demands of online health community users. Recently, more advanced models, such as the Biterm Topic Model (BTM) [35] and the Sentiment-LDA model [36], have been proposed to address these limitations by enhancing topic coherence and incorporating sentiment analysis, respectively.

Within the context of social media’s escalating role in health-related discussions, this study presents an enhanced BERT-LDA model to gain novel insights into users’ informational demands and sentiments. This model, which amalgamates the strengths of the pre-trained BERT model and the LDA model, boosts the utility of topic modeling by harnessing semantic understanding and topic modeling capacities. This method allows for more precise detection of users’ informational demands within online health communities and more sophisticated analysis of emotions inherent in these topics. By offering a thorough evaluation of user information demands, this study bridges a gap in the discipline and contributes to comprehending how social media can influence health policy development. The findings might prove pivotal in molding health policies that are more attuned to the demands and sentiments of the public, as conveyed in online health communities, and provide accurate, tailored information support and services to better meet users’ health information demands.

## 2. Methods

**Figure 1:**
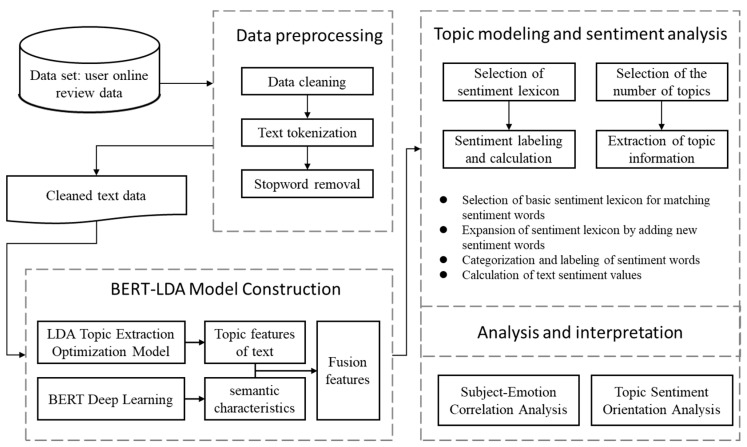
Illustration of the process for topic modeling and sentiment analysis.

The research in this paper follows a systematic approach consisting of four main steps. First, a dataset is constructed and preprocessed, ensuring the relevance and reliability of the data obtained from online health communities. Text mining and topic modeling are then applied in the second step using the BERT-LDA model. This model clusters the text data into different topics, providing statistical analysis of each topic to uncover the main themes discussed in the online health community. The accuracy and reliability of the BERT-LDA model are evaluated and adjusted accordingly. Next, sentiment analysis is conducted in the third step, where a sentiment dictionary is constructed, and a sentiment analysis model is trained. This enables the identification of users’ positive, negative, or neutral sentiments towards each topic. The results of sentiment analysis are visually presented, allowing for a more intuitive understanding of users’ emotional responses. Finally, the findings from topic modeling and sentiment analysis are analyzed and interpreted, providing valuable insights into users’ information demands and emotional responses within online health communities. Patterns, trends, and relationships are identified, and the research outcomes are discussed in relation to existing literature and theoretical frameworks. Recommendations for improving health information delivery and supporting user demands are provided based on the research findings. The specific process is shown in Figure 1.

### 2.1. Dataset Construction and Preprocessing

There are generally two methods for obtaining text data, one is to search for existing datasets, such as third-party corpus libraries like Wiki. The other is to collect data using web crawlers, such as those written using frameworks like Beautiful Soup and Scrapy. For the recognition of information demands of online health communities, open corpora often cannot meet research requirements, so the second method is the main source of obtaining text data. This study uses the well-known domestic online health community “Haodf” as its data source. Currently, online health communities in China can be roughly divided into three categories: medical professional exchange communities (such as DXY Forum), doctor-patient communication communities (such as “Haodf”), and user-initiated discussion communities (such as the Baidu Zhidao Health section). “Haodf” is an online health community established based on user-generated content, encompassing tens of thousands of doctors from hospitals across China, providing patients with online consultations and healthcare services. After receiving online healthcare services, users can share their experiences and express their opinions, viewpoints, and emotions in the form of text comments.

Before actually utilizing text data, it is necessary to clean and preprocess it to make it suitable for the research requirements. As the data collected through web scraping may contain irrelevant information, such as HTML tags, it must be removed to avoid affecting subsequent steps. A limited amount of non-textual content, special non-English characters, and punctuation marks can be deleted using regular expressions in Python.

Tokenization is an important step in text preprocessing, which involves splitting a sentence into multiple independent words. Modern tokenization is based on statistical methods, using samples from standard corpora as the basis for statistical probabilities. By calculating the joint distribution probabilities of different tokenization methods based on the statistical probabilities established from the corpus, the optimal tokenization can be found by selecting the method with the maximum probability for a new sentence. For tokenization demands in text mining, Python components such as NLTK for English tokenization and jieba for Chinese tokenization can be utilized.

The process of removing stop words should be carried out after word segmentation. Stop words are words in a sentence that lack real meaning and do not affect the understanding of the overall semantic meaning of the sentence. In text, there exist a large number of meaningless words such as functional words, pronouns, verbs, and nouns that do not carry specific meanings, for example, “oh” and “ha”. These words do not provide any assistance in text analysis and removing stop words can reduce the workload of subsequent text processing.

### 2.2. Model Construction

#### 2.2.1. LDA Topic Extraction Optimization Model

The Latent Dirichlet Allocation (LDA) is a generative statistical model for discovering the topics that are present in a large corpus of text data. It is commonly used for text data analysis and information retrieval tasks such as document classification and topic modeling [37]. LDA models the text data by assuming that each document is a mixture of several latent topics and that each word in the document is generated from one of these topics. LDA is trained on a large corpus of text data to learn the topics and their distribution and then uses these learned topics to infer the topics for new documents [38].

**Figure 2:**
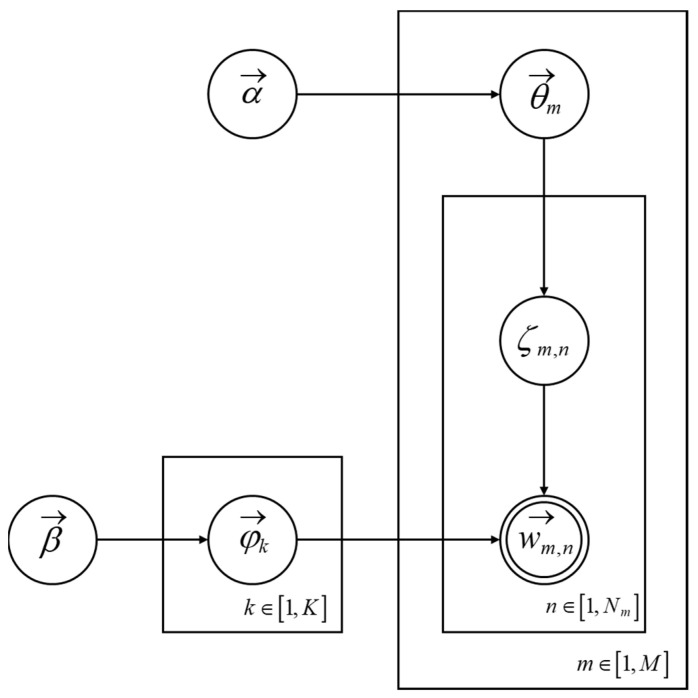
LDA topic analysis model.

As illustrated in Figure 2, the process of $\vec{\alpha }\to {\vec{\theta }}_{m}\to \zeta_{m,n}$ represents the generation of the mth document, where a doc-topic dice ${\vec{\theta }}_{m}$ is first extracted, and then the topic number $\zeta_{m,n}$ of the nth word in the document is generated by throwing the dice. The process $\vec{\beta }\to {\vec{\varphi }}_{k}\to w_{m,n}|=\zeta_{m,n}$ represents the selection of the dice with number $k=\zeta_{m,n}$ from a set of K topic-word dice ${\vec{\varphi }}_{k}$, followed by the generation of vocabulary $w_{m,n}$ through a dice roll. In LDA, the bag-of-words model is also adopted, where M documents correspond to M independent Dirichlet-Multinomial conjugate structures, and K topics correspond to K independent Dirichlet-Multinomial conjugate structures.

Subsequently, parameter estimation was carried out using the Gibbs Sampling algorithm. Iterative sampling was performed until convergence to obtain the “topic-word” distribution matrix φ and the “document-topic” distribution θ. In the original model’s computation process, the topic vector μ was obtained by calculating the cosine distance between the high-frequency words of each topic and the document. However, the topic words were limited by the traditional structure of the LDA bag-of-words model, which cannot effectively combine the semantics and contextual information of the text, and the quality of the word segmentation technique has a significant impact on the calculation results of μ. In order to obtain a better topic vector, this paper integrates the word vectors from the BERT model with the topic representation of the LDA model. By iteratively calculating the word weights, an optimized topic vector μ′ is obtained, aiming to provide more accurate topic semantic information for the sentiment analysis of large-scale complex texts.

#### 2.2.2. BERT Deep Learning Model

**Figure 3:**
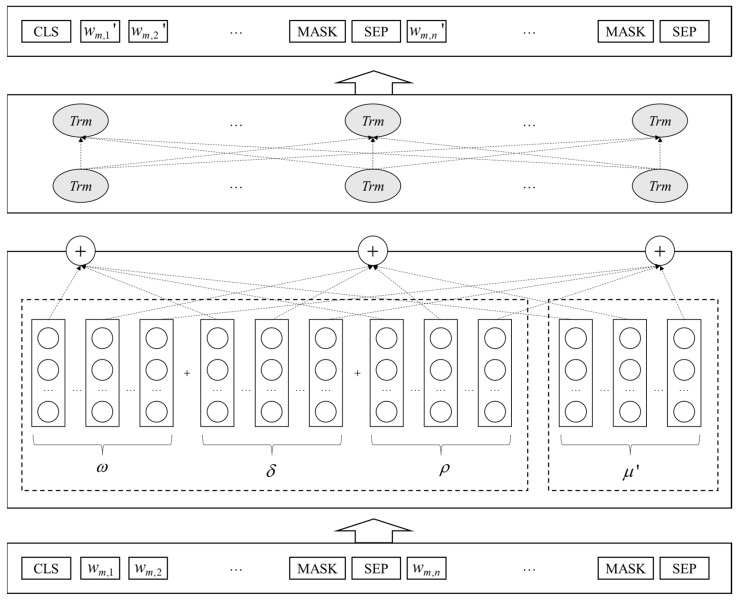
Structure of improved BERT deep learning model.

Bidirectional Encoder Representations from Transformers (BERT), released by Google in 2018, is a language pre-training model based on a bidirectional Transformer architecture [39]. It enhances the model’s semantic representation capabilities through Masked Language Model (MLM) and Next Sentence Prediction (NSP) tasks. By leveraging the powerful feature extraction and fine-tuning transfer learning abilities of Transformers, BERT has excelled in various NLP tasks. To improve the accuracy and granularity of sentiment classification for large-scale complex texts, this paper retains the base BERT model and introduces additional deep pre-training tasks on top of the improved pre-training tasks [40]. Furthermore, the optimized results from the LDA model are embedded into both the pre-training and fine-tuning stages of BERT, allowing the model to learn text features encompassing syntax, semantics, and topics simultaneously during sentiment classification tasks. The structure of the improved BERT deep learning model is illustrated in Figure 3.

Set ω, θ, and ρ to represent the word, document, and position vectors of the model when processing text, respectively. Trm denotes the Transformer encoder unit, and $d_{i}\prime$ refers to the collection of word vectors for document $d_{i}$ that have been enhanced with improved full-text semantic information. For special characters inserted into the text, CLS represents the text start symbol, SEP denotes the text separator and end symbol, and MASK signifies the masking character. As shown in Figure 3, after tokenizing and inputting the document into the model, each word is mapped to three vectors and represented as $w_{m,n}(\omega +\theta +\rho )$, collectively referred to as word vectors. In the improvement process, the word vector $w_{m,n}(\omega +\theta +\rho )$ is combined with the LDA-optimized topic representation, resulting in a word vector $w_{m,n}(\omega +\theta +\rho +\mu \prime )$ with enhanced topic vectors. This is then fed into a bidirectional Transformer encoder. To enable the model to learn more information, the Transformer encoder connects the multi-head mechanism and feed-forward layers through a residual network structure [41]. The multi-head mechanism applies multiple linear transformations to the input vector, obtaining different linear values and subsequently calculating attention weights.

In this way, the Transformer encoder learns and stores the semantic relationships and syntactic structure information of document $d_{i}$. As document $d_{i}$ has been enhanced with superior topic feature vectors through the BERT model improvement, the improved document $d_{i}\prime$ is connected to the softmax output layer via the special character CLS to adapt to transfer learning under multiple tasks. Thus, the integration of topic-optimized feature vectors and BERT word vectors is expected to improve the accuracy and granularity of sentiment analysis in large-scale complex texts.

### 2.3. Sentiment Analysis

Online health community sentiment analysis involves employing natural language processing techniques to analyze the emotions present in posts or comments within an online health community. This enables individuals to gain insights into opinions and emotions regarding health issues, with the aim of providing more effective health treatment and support [42]. The fundamental principle includes preprocessing the text through techniques such as vocabulary analysis, part-of-speech tagging, tokenization, and removal of stop words. Subsequently, sentiment dictionaries, machine learning models, or correlation analysis are employed to evaluate the emotional inclination of the text, determining whether it is positive, negative, or neutral.

**Figure 4:**
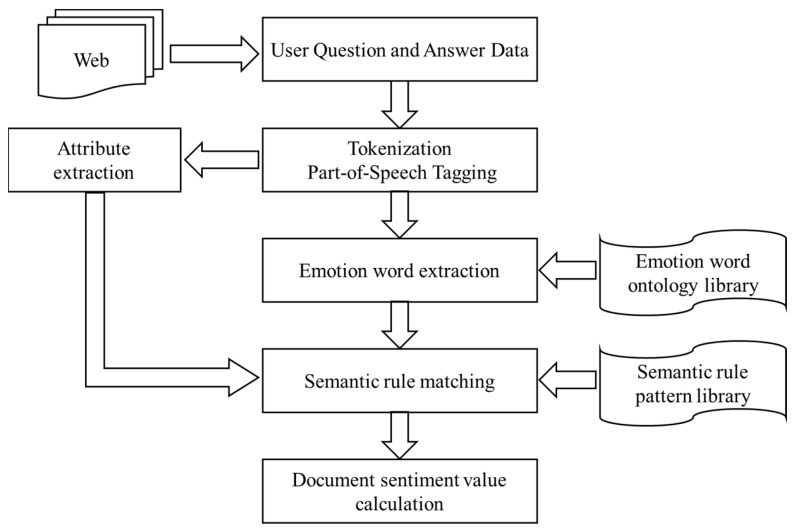
The calculation process for sentiment value.

This paper utilizes the Chinese sentiment ontology constructed by Dalian University of Technology to analyze the relationship between the topics of online health community user information demands and emotions. All emotions are divided into seven categories: “Joy”, “Good”, “Anger”, “Sadness”, “Fear”, “Evil”, and “Surprise”, and corresponding 21 subcategories. The sentiment value in this paper is set to 0, 1, −1, 3, representing neutral, positive, negative, and ambivalent, respectively. If a sentiment word has two identical polarities, such as both 1 or -1 or 0, then the sentiment value is determined by the first [intensity, polarity]. If a sentiment word has two identical polarities and both are 3, then the polarity of the sentiment value is determined by the sum of the polarity of the sentiment value of the previous 0-4 and the polarity of the sentiment value of the latter 0–4, multiplied by 0.75 (the ratio of previous and later can be modified), and calculated only for 0, 1, −1. Finally, the result is determined by the absolute distance from −1, 0, 1, and the correlation between each information demand topic and its sentiment is analyzed. The calculation process for sentiment value is illustrated in Figure 4.

## 3. Result

In this study, we utilized the BERT-LDA model for topic discovery and empirical analysis, training it directly on Chinese textual data and bypassing the need for translation into English. This methodology was chosen to preserve the subtle nuances and context embedded in the original Chinese text, potentially compromised during translation. However, due to intricate grammatical structures and unique characters in the Chinese language, this presented distinctive challenges. For the BERT model, we selected BERT-base as the foundational model. The pre-trained weights were fine-tuned to adjust to the task of topic discovery within Chinese online health communities, a process requiring a careful balance between preserving the model’s pre-existing knowledge and enabling it to adapt to the task-specific nuances. The BERT model was further utilized for a text classification task, employing its contextual understanding to accurately pinpoint topics and sentiments in the Chinese textual data. Implementation of the LDA model necessitated setting the number of topics to 36, based on perplexity and coherence calculations. Over the course of 1000 iterations, the model’s topic and word distributions were optimized to yield precise topic discovery results. The model’s hyperparameters, specifically alpha and beta, were assigned values of 1.0 and 0.01 respectively, ensuring suitable sparsity and smoothness. During data preprocessing, the raw Chinese text underwent cleansing and tokenization, and a bag-of-words model was implemented to represent the text. For model training and evaluation, the dataset was split into training and validation sets in an 80:20 ratio. Evaluation metrics such as accuracy gauged the model’s performance in topic discovery. Despite the inherent challenges of training the model directly in Chinese and the substantial computational resources required, this method proved successful, as demonstrated by the accurate topic discovery and sentiment analysis results achieved. Consequently, these parameter settings established a robust foundation for empirical analysis, with the ultimate objective of accurately identifying topics and sentiments within the text data.

### 3.1. Data Collection and Preprocessing

There are two main reasons for selecting “Haodf”: first, it has a high degree of recognition, a large user base, and a high daily activity level, providing abundant and real-time updated online consultation data; second, as a doctor-patient communication community, “Haodf” has a large number of real-name certified doctors, and the professional level of online consultations is relatively high. Therefore, this study selects user question-and-answer data from September 2021 to September 2022 as experimental samples and utilizes Python web scraping to obtain the Q&A records from this online health community. After eliminating invalid comments and deleting duplicate comment data, a total of 10,728 comments were collected for topic mining and sentiment analysis.

### 3.2. Health Information Demands Topic Identification

**Figure 5:**
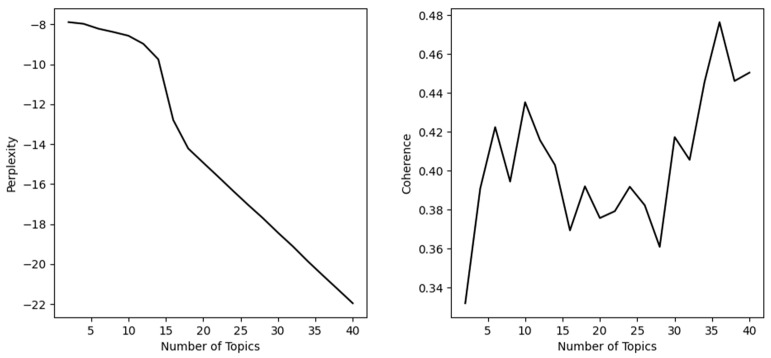
Changes in perplexity and coherence with the number of topics.

In topic modeling, perplexity and coherence are commonly used to evaluate the quality and interpretability of models. Perplexity is a metric used to measure a model’s ability to fit new text data, with lower perplexity indicating better predictive capabilities. Coherence, on the other hand, is used to assess the interpretability of the topics generated by a topic model, with higher coherence indicating better quality of generated topics. Generally, perplexity decreases with an increasing number of topics, while coherence initially increases and then decreases with the number of topics. Therefore, when selecting the optimal number of topics, it is necessary to consider the changes in perplexity and coherence together. The optimal number of topics should have both low perplexity and the highest point of coherence. This approach helps avoid overfitting issues while improving the quality and interpretability of the topic model. Figure 5 in this paper illustrates the changes in perplexity and coherence with the number of topics. Based on the aforementioned analysis, when the number of topics is set to 36, the perplexity is relatively low, and coherence is the highest. Thus, we choose K = 36 as the optimal number of topics.

**Figure 6:**
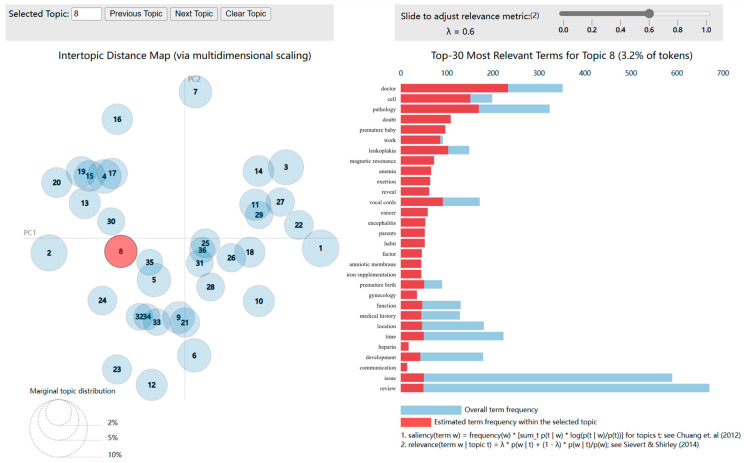
Visual analysis of topic clustering [43,44].

The BERT-LDA model was employed to identify topics, and topic clustering visualization was achieved through the pyLDAvis visualization tool. In the topic clustering visualization results, the bubbles on the left represent topics, with their size indicating the frequency of occurrence. The farther apart the bubbles are, the lower the similarity between the corresponding topics. overlapping bubbles indicate the presence of shared characteristic words between topics. On the right side, the top 30 characteristic words within each topic are displayed. The light blue color represents the frequency of occurrence of each word in the entire document, while the deep red color represents the weight of each word within the specific topic. Adjacent to the right, an adjustable parameter, denoted as λ, is available to modify the relevance between words and topics. When the value of parameter λ approaches 0, words that are more unique to the topic become more relevant. Conversely, when λ approaches 1, words that appear more frequently within the topic become more relevant. By summarizing the meaning conveyed by these vocabulary terms and their correlation with the corresponding topics, the significance of each topic can be deduced. Please refer to Figure 6 for a detailed illustration.

In the process of performing topic modeling on the initial dataset, a set of topic probability distributions can be obtained. By considering the probability distribution of each document across all topics, each document can be assigned to the topic with the highest probability, thereby determining the topic most relevant to the document. Therefore, in this study, the topic with the highest probability is considered as the document’s primary topic and serves as the basis for its final classification. The final model yielded 36 topics after training, and the contents of these topics are shown in Table 1. From Table 1, it can be observed that relevant vocabulary is clustered within the same topic, indicating a good clustering effect of the topic model.

By employing the BERT-LDA model, this study categorized words into topics based on their co-occurrence within the dataset. Notwithstanding its efficacy in pattern detection, the model might group seemingly disparate words, medically speaking, under the same topic due to their co-occurrence frequency. A case in point is the combination of ‘chemotherapy’ and ‘nasal congestion’ under ‘Q7 Prevention’. Although seemingly counterintuitive, this outcome stems from the model’s focus on pattern detection, which might not consistently align with prevailing medical knowledge. It becomes apparent that exclusive reliance on topic modeling for categorizing health-related information from online communities may not yield perfect accuracy. To counteract this issue, the study incorporated relevant references from the literature [45,46,47]. Finally, through the analysis of visualized results (Figure 6), the initial 36 topics were categorized into nine major topics: Causes, Symptoms and manifestations, Examination and diagnosis, Treatment, Self-management and regulation, Impact, Prevention, Social life, and Knowledge acquisition (Table 2). These topics represent the most common topics found in disease-related treatment information and reflect the most important information within disease-related discussions.

### 3.3. The Sentiment Analysis of Health Information Demands Topics

In order to improve the accuracy of text segmentation for posting and reply, a custom base dictionary is first constructed. The custom base dictionary includes a basic sentiment dictionary and a medical field dictionary. The basic sentiment dictionary selects 27,466 sentiment words from the sentiment ontology library of the Dalian University of Technology, which categorizes sentiment into seven major categories and 21 subcategories, with sentiment intensity ranging from weak to strong, divided into five levels of 1, 3, 5, 7, and 9. The sentiment classification meets the requirement of detailed sentiment category analysis. The medical field dictionary is processed and imported into the custom base dictionary through the Medical Dictionary, and then into the segmentation program to segment the preprocessed text. The recognized words are then matched with sentiment words in the sentiment ontology library of Dalian University of Technology and sentiment words are extracted. According to the corresponding relationship between sentiment words and the sentiment ontology library of Dalian University of Technology, the sentiment category and intensity of each word are determined, and the sentiment category and intensity of each sentence are then calculated based on this information.

#### 3.3.1. Subject–Emotion Correlation Analysis

**Figure 7:**
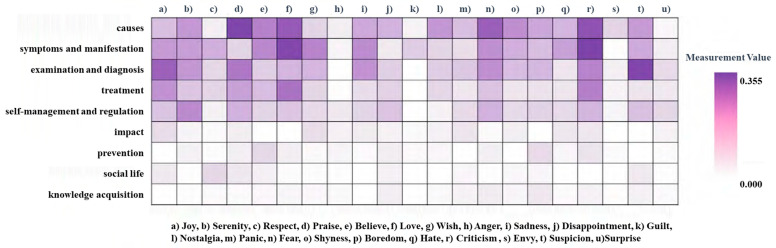
Topic–emotion correlation heat map of information demands.

In this study, the Pearson correlation analysis method was used to delve into the relationship between the various health information demand topics and emotions. The correlation coefficients between the 9 topics and 21 emotions were calculated, and when *p* < 0.05, it was considered that there was a significant correlation between the variables. Based on the correlation coefficients, a heat map of the information demand topic–emotion correlation was drawn, as shown in Figure 7. It clearly displays the pattern between the health information demand topics and 21 emotions among users of online health communities. The color of the square in the figure represents the size of the correlation coefficient between the topic and emotion corresponding to the row and column, and the deeper the color, the more significant the correlation between the topic and emotion, and the closer to white, the weaker the correlation.

Based on this, the correlation between information demands and emotions can be divided into three categories. The first category has a significant correlation with the majority of emotions and a high correlation with some emotions, including “self-management and regulation”, “social life”, and “impact”. The second category also has a significant correlation with various emotions, but the correlation is general and more average, including “examination and diagnosis” and “prevention”. The third category is almost devoid of emotional bias, and the correlation with all emotions is weak or even unrelated, including “symptoms and manifestations”, “causes”, “treatment”, and “knowledge acquisition”.

From an emotional standpoint, numerous emotions manifest in the process of conveying information demands by healthy community members, while a few emotions, such as “anger”, “guilt”, “jealousy”, and “surprise”, exhibit no significant association with all topics. For topics exhibiting higher emotional correlations, distinct topics and emotions demonstrate varying degrees of correlation. Topics such as “praise”, “fear of approval”, “criticism”, and “self-regulation” are closely linked, as these focus on users’ emotional expression, resulting in a balance of positive and negative emotions concerning emotional correlation. The topics of “approval”, “criticism”, and “social life” are highly related, displaying clear emotional expression. Several factors contribute to these observations. Thus, on one hand, users exhibit positive and optimistic emotions due to the improvement in their condition and social relationships. On the other hand, they may experience critical emotions arising from their own illness or that of friends and family, with the majority of this criticism manifesting as self-blame, and minimal criticism or devaluation directed towards family, friends, or medical professionals. The generation of critical emotions primarily stems from two situations: first, patients enduring long-term illnesses feel as though they are burdening their families, and second, pathological self-blame originating from mental illness. The correlation levels between “influence” and “happiness” and “doubt” indicate patients’ ambiguous and skeptical perspectives of their own value.

#### 3.3.2. Topic Sentiment Orientation Analysis

**Figure 8:**
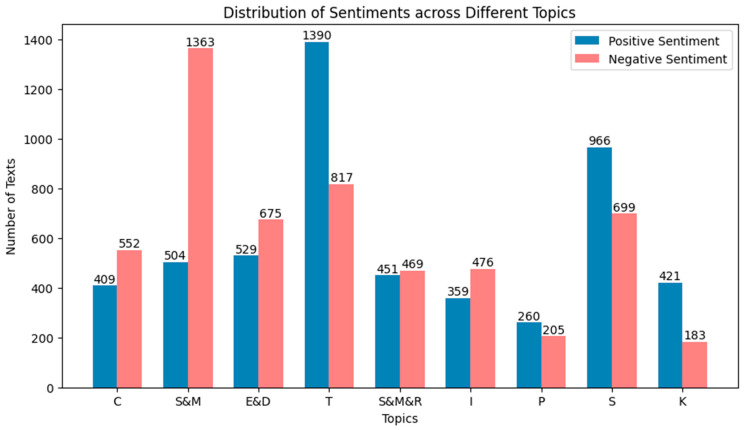
Distribution of positive and negative sentiment across different topics.

This study analyzes the emotional tendencies of texts across various topics, with a total of 10,728 health information demand texts divided into nine categories. The distribution of positive and negative texts for each topic is presented in Table 3 and Figure 8. There are differences in positive and negative texts across different topics. Among the documents, 63% of the texts on the treatment topic have a positive emotional tendency, while 37% have a negative tendency. In the prevention topic, 56% of the texts have a positive tendency, and 44% have a negative tendency. This indicates that people tend to express more positive emotions in treatment and prevention topics. However, in the symptoms and manifestations topic, only 27% of the texts have a positive tendency, and 73% have a negative tendency, which may be due to the involvement of physical discomfort and health issues, causing people to express negative emotions more easily. In addition, the distribution of emotional tendencies in other topics was observed. For example, in the self-management and regulation topic, 49% of the texts have a positive tendency, and 51% have a negative tendency. In the social life topic, 58% of the texts have a positive tendency, and 42% have a negative tendency. These data reveal differences in people’s emotional experiences of health information across different topics. These findings serve as a reminder that health information provision should pay attention to the diversity of user emotions and provide comprehensive support. Positive emotional expressions can enhance user confidence and motivation, aiding them in better coping with health issues. Additionally, the expression of negative emotions should also be given due consideration, in order to provide emotional support and coping strategies for users.

### 3.4. Model Comparison Analysis

To validate the reliability and advantages of the proposed method, this study compares the performance of the LDA model, Word2Vec model, BERT model, and the BERT-LDA model used in this paper. A ten-fold cross-validation method is employed for training and testing the dataset, resulting in F1 scores of 81.2%, 85.5%, 89.5%, and 93.4%, respectively. The recognition results are shown in Table 4.

As shown, the BERT-LDA model achieves the best topic recognition performance, with precision, recall, and F1 scores all higher than the other models. This model can learn syntactic, semantic, and topic features of the text simultaneously when performing classification tasks, overcoming the issue of missing text semantics to a certain extent. It can more comprehensively and accurately represent the semantic information of text vectors, improving the model’s accuracy and granularity in large-scale complex text sentiment analysis. This validates the effectiveness of the proposed model.

## 4. Discussion

This paper employs text mining methods to investigate the health information demands of users in online health communities. The content characteristics, emotional characteristics, and underlying reasons for various health information demands are analyzed.

(1) This research paper utilizes text mining techniques to investigate the specific health information demands of users in online health communities. The study analyzes the characteristics of the content and emotions expressed in these information demands, as well as the underlying reasons behind them. The findings of the study reveal that users in online health communities have a wide range of health information demands, which can be classified into nine main topics. These topics include causes of illnesses, symptoms and manifestations experienced, examinations and diagnoses, treatment options, self-regulation techniques, the impact of the illness on their lives, prevention strategies, social aspects related to their health, and acquiring knowledge about their condition. The study further highlights that users’ information demands primarily revolve around physical sensations, physiological changes, emotional fluctuations, and information related to their treatment options. However, they show less interest in general health information, such as overall health knowledge and disease prevention. When it comes to topics dominated by symptoms and causes, users tend to express their information demands in an objective manner, using professional terms like examination indices and disease names. Their language is concise, direct, and lacks strong emotional features. On the other hand, topics related to life and emotions reveal a greater emotional diversity among users. In these topics, users use more casual and colloquial language, provide more detailed descriptions, and express a wider range of emotions. As a result, the study finds that users’ emotional expressions exhibit a characteristic of centrality across different topics. This means that most emotions are concentrated and significantly correlated with only a few specific topics.
(2) The examination of user sentiment within online health communities discloses a pronounced predominance of negative emotions. As the study substantiates, this emotive climate significantly shapes users’ information-seeking behaviors, engendering a distinctive nexus between emotions and information demand. Negative emotions, frequently serving as a catalyst, propel users towards the consumption of health-related information. Intriguingly, the study also detects a crucial component of support-seeking behavior within these communities. Users exhibit a noticeable inclination for supportive and uplifting sentiments, highlighting the emotional role these online communities fulfill in tandem with their function as health information platforms. This intricate interconnection between users’ information demands, their emotional state, and the supportive environment they aspire to foster within online health communities accentuates the multifaceted nature of these platforms. Their role transcends the bounds of mere repositories of health information, morphing into a sanctuary offering empathy and emotional succor. This revelation necessitates a reassessment of strategies for managing and moderating online health communities, suggesting an increased need for empathetic and emotionally intelligent AI models, alongside personalized health information services. Acknowledging the emotional undercurrent in the information-seeking process and provisioning an environment responsive to this requirement could be instrumental in bolstering user engagement and enhancing the overall user experience within online health communities.
(3) Within online health communities, users frequently confront numerous challenges in their pursuit of disease-related information. The current dissemination of disease-associated knowledge is somewhat constrained, resulting in a narrow understanding of relevant illnesses among community members. Therefore, patients’ information requirements typically gravitate towards symptoms and diagnostic results pertinent to their conditions. Regarding emotional and psychological issues potentially implicating personal privacy, users have demonstrated a preference for leveraging online health communities [48,49], as evidenced by data harvested from the “Haodf” platform. However, the information-seeking journey within these communities is not devoid of difficulties. Users regularly grapple with an overwhelming abundance of data related to obscure illnesses, which presents a significant obstacle to the pinpointing of germane information. Inquiries lodged by users often go unaddressed or receive contributions from non-expert sources, leading to the proliferation of unverified information. Additionally, users’ shared experiences with various diseases frequently go unnoticed, culminating in incomplete information. As users seek disease information, they often encounter issues such as nebulous sources, insufficient evidence, misinformation, or even malevolent rumors. These factors precipitate information confusion and undermine reliability, thwarting the complete integration and utilization of the vast resources accessible within online health communities. Nevertheless, despite these impediments, the preference for online health communities for information procurement, especially in privacy-sensitive matters, underscores the pivotal role these platforms occupy in health information dissemination [50,51].
(4) The findings of this study are in alignment with existing literature in several aspects, providing a deeper interpretation through the comparison and contrast with previous research. Consistent with studies that focus on online health communities and social media [20,21,22,46], this study explores the impact of individual factors, such as motivation, on users’ willingness to obtain and distribute health information. The results confirm that users primarily seek information related to their specific conditions, such as symptoms, examination results, and treatment options while showing less interest in general health information like disease prevention. This study’s findings are in line with Liu et al.’s research, which found a slight positive relationship between the number of users expressing negative emotions and the size of an online health community. This suggests that negative emotions play a significant role in driving the demand for information in these communities. Additionally, the results align with previous studies on Facebook, which examined the types of users seeking emotional support and highlighted the positive effects of such support. Like these studies, our findings indicate that users prefer to receive supportive and uplifting emotions through online health communities. Moreover, the sentiment tendencies identified in this study are similar to those found by Vydiswaran et al. [26]. This highlights the significance of understanding the connection between users’ health demands and emotional demands in order to improve the efficiency of information retrieval and explain information behavior. In conclusion, this study demonstrates that the results align with previous research and emphasizes the demand to take into account both individual factors and emotional expression when addressing the information demands of users in online health communities.

## 5. Conclusions

### 5.1. Possible Research Contributions

This research contributes to the field of online health community user information demands in several ways. Firstly, it offers a comprehensive understanding of online health community users’ information demands by analyzing a large dataset of user-generated content. The identification and analysis of the nine major categories of health information demands contribute to a more nuanced understanding of users’ information-seeking behaviors and preferences.

Secondly, the incorporation of sentiment analysis into the topic modeling framework introduces a new dimension to the analysis. By considering the sentiment associated with each topic, researchers and practitioners can gain insights into the emotional aspects of user information demands. This knowledge can inform the development of more targeted and empathetic health information services that take into account users’ emotional states and provide appropriate support.

Moreover, the proposed BERT-LDA model presents an enhanced approach to topic modeling in the context of online health communities. By leveraging BERT’s semantic understanding and LDA’s topic modeling capabilities, the model achieves more accurate topic identification and sentiment analysis. Integrating the BERT-LDA model into the topic modeling process, this research contributes to a more precise and comprehensive analysis of users’ information demands and sentiments in online health communities. The systematic approach employed in this study provides a solid foundation for future research in the field of online health community analysis.

### 5.2. Limitations and Future Research Directions

While this research provides valuable insights into the informational demands and sentiments of online health community users, it is not without its restrictions. These primarily stem from the data source utilized. The analysis was restricted to textual data harvested from a single online health community, which may not fully encapsulate the diversity of users and their information needs across different platforms or offline environments. Incorporating varied data types, such as user demographics, engagement metrics, and social network structures, could enhance future research. This addition would provide a more thorough comprehension of users’ informational demands and preferences. The sentiment analysis within this study was primarily at the topic level. By expanding sentiment analysis to the document or sentence level in future research, a more nuanced interpretation of the emotions and attitudes expressed by users could be attained. Regarding model enhancement, the proposed BERT-LDA model, though sophisticated, still holds potential for further refinement. Future studies could experiment with hybrid models or variations, incorporating multiple cutting-edge natural language processing techniques. These advancements could mitigate the inherent limitations tied to topic modeling and sentiment analysis, thereby improving the model’s precision and effectiveness. Furthermore, this study did not examine the influence of diverse factors like user characteristics, cultural backgrounds, and health literacy levels on online health community users’ informational demands and sentiment expressions. Future research could investigate these aspects to foster the development of more personalized, culturally sensitive health information services.

In conclusion, while this research has made significant contributions to understanding online health community user information demands and sentiment, there are still opportunities for further research. By addressing the aforementioned limitations and exploring new avenues of investigation, future studies can contribute to the advancement of knowledge in this field and enhance the provision of tailored and effective health information services in online communities.

## Figures and Tables

**Table 1:** Distribution of user information demands in online health communities.

Topic	Top 5 High Probability Keywords	Topic	Top 5 High Probability Keywords
Topic 1	Chemotherapy, Over a month, Nasal congestion, Runny nose	Topic 19	Hospitalization, Adjustment, Diabetes, Phone call, Patients
Topic 2	Body, Abdomen, Solutions, Cervix, Joints	Topic 20	Epidemic, Outpatient, Report, Myoma, Taking medication
Topic 3	Ears, Complete blood count, Location, Issues, Lymph nodes	Topic 21	Follow-up examination, Examination, Timing, Fetal bud, Condition
Topic 4	Symptoms, Common, Abdominal pain, Discharge, Brownish	Topic 22	Attack, Indicators, Doctor, Urine protein, Kidney disease
Topic 5	Skin, Hyperthyroidism, Medication, Diseases, Fingers	Topic 23	small blister, Red, Child, Etiology, Tongue
Topic 6	Pain, Patients, Guidance, Unable to, Triggers	Topic 24	Plan, Surgery, Examination, Hepatitis B, Family members
Topic 7	Results, Asthma, Hypertension, Blood lipid, Calcium supplements	Topic 25	Eczema, Anus, Prostatitis, Lymph nodes, Buttocks
Topic 8	Doctor, Cell, Pathology, Doubt, Premature baby	Topic 26	Rash, Doctor, Red rash, Nerves, Weight, Endocrine
Topic 9	Glass, Month, Nodules, Ovaries, Whole body	Topic 27	Condition, Doctor, Pop-up window, Blood pressure, Severity, Stress
Topic 10	Results, Examination, Father, Affected child, Hours	Topic 28	Hospital, Feeling, Patient, Vitiligo, Laryngoscope
Topic 11	Nodules, Patients, Lungs, Headache, Films	Topic 29	Bladder, Sperm, Test tube, Chin, Syndrome
Topic 12	Child, Fracture, Positive, Patients, Pain	Topic 30	Medication, Influence, Rhinitis, Child, Taking medication, Feeling
Topic 13	Examination, Recommendation, Patients, Face, Lips	Topic 31	Disorder, Eyes, Traditional Chinese medicine, Emotion, Bipolar
Topic 14	Causes, Testing, Discharge, Professor, Development	Topic 32	Consultation, Question, Doctor, Cyst, Throat
Topic 15	Menstruation, Vagina, Lower abdomen, Quantitative, pain	Topic 33	Time, Doctor, Menstruation, Lump, Endometrium
Topic 16	Medication, Recommendation, Doctor, Mosquitoes, Severe	Topic 34	Dizziness, Patients, Heart, Prescription, Weakness
Topic 17	Doctor, Condition, Surgery, Recommendation, Medication	Topic 35	Surgery, Patients, Breast cancer, Nose, Tumor
Topic 18	Pneumonia, Mucus gland carcinoma, Bronchus, Child, Constitution	Topic 36	Lump, Redness, Effect, Acne, Location

**Table 2:** Categorized topics in online health communities.

Category	Topics	Description	Number of Documents	Proportion (%)
Q1 Causes	4, 15, 17, 19	Ask about the causes of a disease or behavior	961	8.96
Q2 Symptoms and manifestations	18, 25, 26, 28, 31, 36	Ask about the symptoms and manifestations of a disease	1867	17.40
Q3 Examination and diagnosis	9, 21, 32, 33, 34	Ask if a disease is present and what type of disease it is Ask about the approach, method, or effectiveness of a test for a disease	1204	11.22
Q4 Treatment	2, 8, 8, 24, 35	Ask if you should see a doctor or ask for a referral Ask if a treatment is effective and if treatment is needed Ask about other treatment options	2207	20.57
Q5 Self-management and regulation	13, 20, 30	Ask how to manage and regulate negative emotions caused by illness	920	8.58
Q6 Impact	6, 12, 23	Ask how an illness affects children, spouses, parents Ask how to avoid the impact	835	7.79
Q7 Prevention	1, 10	Ask how to prevent or avoid a disease problem	465	4.33
Q8 Social life	3, 11, 14, 22, 27, 29	Describe health problems of parents, relatives and children How do you get along with them Ask for the community’s assessment of the phenomenon, behavior, or event resulting from the disease Describe your health problems and ask for understanding and support	1665	15.52
Q9 Knowledge acquisition	7, 15	Ask about disease and health	604	5.63

**Table 3:** Detailed topic sentiment orientation analysis with additional sentiment words.

Topic	Number of Documents	Positive Words	Positive Sentiment	Negative Words	Negative Sentiment
causes (C)	961	praise, encouragement, support…	409 (43%)	blame, worry, suffering…	552 (57%)
symptoms and manifestations (S&M)	1867	comfort, improvement, relief…	504 (27%)	pain, severity, distress…	1363 (73%)
examination and diagnosis (E&D)	1204	accurate, professional, efficient…	529 (44%)	delay, misdiagnosis, inaccuracy…	675 (56%)
treatment (T)	2207	effective, significant, innovative…	1390 (62%)	failure, side effects, expensive…	817 (38%)
self-management and regulation (S&M&R)	920	self-discipline, progress, control…	451 (49%)	struggle, indulgence, difficulty…	469 (51%)
impact (I)	835	positive, change, adaptation…	359 (43%)	negative, destruction, barrier…	476 (57%)
prevention (P)	465	effective, prevention, advance…	260 (56%)	neglect, disregard, oversight…	205 (44%)
social life (S)	1665	integration, friendship, support…	966 (58%)	isolation, discrimination, conflict…	699 (42%)
knowledge acquisition (K)	604	learning, improvement, comprehension…	421 (70%)	confusion, misinformation, misunderstanding…	183 (30%)

**Table 4:** Model comparison.

Model Type	Precision	Recall	F1 Value
LDA Model	0.821	0.803	0.812
Word2Vec Model	0.863	0.848	0.855
BERT Model	0.909	0.882	0.895
BERT-LDA Model	0.945	0.923	0.934

## Data Availability

The dataset presented in this research is available with a legitimate request from the corresponding author.
